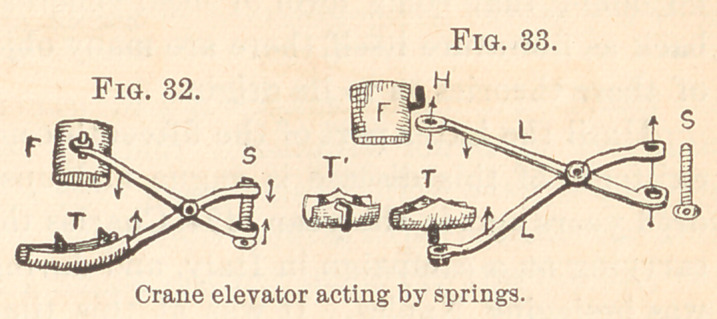# Elevation of Teeth in the Sockets

**Published:** 1894-07

**Authors:** J. N. Farrar

**Affiliations:** New York City


### International Dental Journal.

Vol. XV. July, 1894. No. 7.

### Original Communications.[1]

### ELEVATION OF TEETH IN THE SOCKETS.

BY J. N. FARRAR, M.D., D.D.S., NEW YORK CITY.

(Continued from page 211.)

Elevation of the teeth, or, as it is sometimes erroneously called, "elongation" of teeth, has in the past been regarded a hazardous operation, because so slight force is necessary to move them, that they are liable to be forced too far from their former places. Twenty-five years ago the plan of operation was to tie sheet-rubber to the neck of the "short tooth" in such a way as to draw upon it by being stretched over the ends of the adjacent teeth. In 1879, Dr. A. N. Chapman hit upon the greatly-improved plan of tying a string about the neck (under the gum) of the tooth to be elevated (or to a knot on a wire bound around the neck), and then drawing the extremities of the string over a stiff piece of wire extending from over the end of one adjacent tooth to, and over, the end of the opposite adjacent tooth, the wire being held on these adjacent teeth by two small saddles of gold plate, one of which was soldered to each extremity. The only defect in this plan was that there was nothing but the loop of the draught-string to prevent the tooth from being drawn too far. This defect I overcame by soldering a third saddle

[1] The editor and publishers are not responsible for the views of authors of papers published in this department, nor for any claim to novelty, or otherwise, that may be made by them. No papers will be received for this department that have appeared in any other journal published in the country.

(or a trough) to the middle of the bridge-wire, for lodgement of the moving tooth when it is drawn to its proper place.

To perform these operations more easily, I have devised several mechanisms, some acting by elastic rubber, some by wire springs, and others by screws. Some of these are only modifications of the Chapman mechanism. Omitting those that are similar to Chapman's, and passing over most of those acting by elastic rubber and by metallic springs, a few will be presented without going into details of the operations.

Fig. 19 represents a mechanism acting by elastic rubber, designed for elevating a left upper lateral incisor. This consists of a rubber ring, R; a piece of platinum wire, C; clamp-band, B, having a wire arm, W, projecting from its anterior part, and having soldered to it two troughs, T, K, and three hooks. In applying this mechanism, the wire C is first made to encircle tightly the neck of the tooth to be elevated, leaving a *twisted knot* on the labial side. The clamp-band is then fastened on the left bicuspids, leaving the trough, or saddle, T, to ride on the end of the left central. After this, the rubber ring is caught on the knot on the wire C, and stretched through the hook under the lateral, thence to and caught on the hook near the anchor band.

FIG. 19.

Elevating mechanism acting by elastic rubber ring (*A*).

*Wire Spring.*—Figs. 20 and 22 represent two modifications of mechanism acting by wire springs. Fig. 21 represents application of Fig. 20.

Each of these consists of two springs (S), a ferrule for the "short tooth," and either a saddle, or three caps, on the sides of which the

FIG. 20.

FIG. 21.

FIG. 22.

Elevators acting by springs.

springs are attached (by soft solder). Having cemented the ferrule on the "short tooth" and placed the saddle on the edges of the front upper teeth, the springs are caught on the hooks on the ferrule. When the teeth are crowded, a piece of platinum wire bound around the neck of the tooth to be elevated is preferable to a ferrule, because it does not increase the crowd.

Fig. 23 illustrates an operation for elevating a right upper lateral. The mechanism consists of a ferrule clamp-band, B, and a wire hair-pin-shaped spring soldered to the posterior nut. On the extremity of the lower arm is soldered a saddle, T, to rest on the end of the right central. The other arm rests on a hook on a ferrule, F, on the lateral. The tooth is acted upon by a sliding ferrule, O.

Fig. 23.

Operation by an elevator acting by a spring.

Fig. 24.

Operation by an elevator acting by a sliding nut.

*Screw-acting.*—Fig. 24 illustrates the application of a similar mechanism. Fig. 25 illustrates a similar operation and a similar mechanism, but acting by a screw instead of a sliding nut. This

Fig. 25.

Operation by an elevator acting by a screw.

Fig. 25′.

The mechanism.

mechanism, represented separately by Fig. 25′, is, in my opinion, the best mechanism ever devised for elevating a tooth in its socket. This one, which was for elevating a right upper lateral, consisted of a clamp-band, B, and a screw, S, having two wire arms, A, A, with saddles, C, V, a collar wire, W (platinum), for the neck of the lateral to be elevated. In applying the mechanism, the wire W is first made to encircle tightly the neck of this lateral, leaving the knot (twisted) on the labial side for one of the arms to ride on. The anchor-band is then fastened on the right bicuspids, leaving the saddle C to rest on the end of the right central and the saddle V directly under the lateral, but not in contact with it. This mechanism is operated by turning the screw S so that its head forces the upper arm A downward, as indicated by an arrow. To insure against depression of the central it is sometimes well to have its saddle rest on both centrals.

Figs. 26 and 27 represent a mechanism for an operation for elevating a right lower lateral. The mechanism consists of a clamp-

FIG. 26.

FIG. 27.

Elevators acting by screws.

band, two screws, two wire arms, and a ferrule. The wire arms, which are attached by a rivet to an eye below the anterior clamp-band nut, are operated upon by a screw in the double posterior nut, pushing along the wires a flange nut riding on the collar between the screw and its key nib.

Figs. 28 and 29 illustrate an operation and mechanism for elevating left upper lateral. This hair-pin mechanism is made similar

FIG. 28.

FIG. 29.

Elevators acting by screws.

to the one represented by Fig. 26, the difference being mainly in the attachment of the wire arms W, W to the posterior nut of the clamp-band B, being made by solder instead of a rivet. The screw S, by pushing the flange nut N along the wires, as shown, causes the extremities to approach each other.

Figs. 30 and 31 illustrate an operation for elevating a left upper cuspid by scissors-shaped arms, W, W, operated by a screw, S. The

FIG. 30.

FIG. 31.

jointing and anchoring of the arms is made by a rivet through two ears, E, soldered to the anterior nut of the clamp-band B.

Figs. 32 and 33 represent my crane elevator. After the broad ferrule F is cemented (with phosphate of zinc) to the tooth to be elevated, the upper arm L is caught on the hook H. The trough T is next placed on the ends of the teeth adjacent to the one to be acted upon, and then the screw S is placed in the holes in the other ends of the arms, which are then tightened. The crane-like arms are then swung into the mouth and left behind the lip or cheek-tissues. This mechanism is practicable, but too fancy to be equal to the one represented by Fig. 25, which is the best of all these my inventions.

## Figures and Tables

**Fig. 19. f1:**
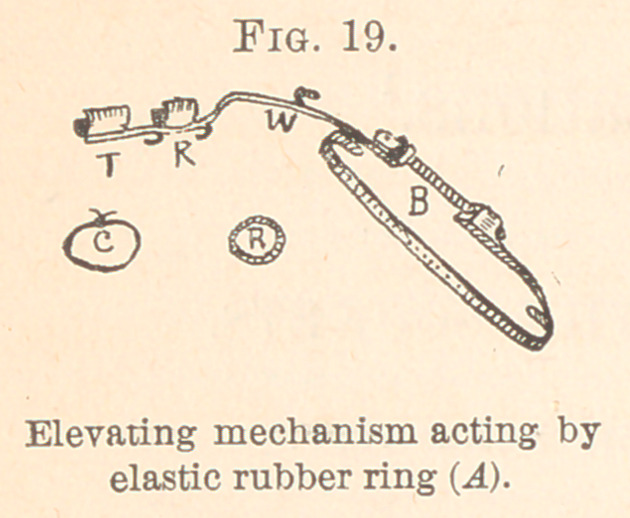


**Fig. 20. f2:**
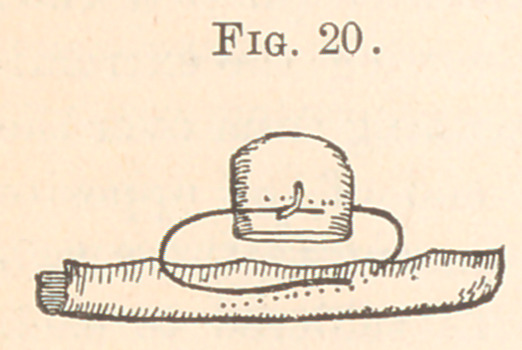


**Fig. 21. f3:**
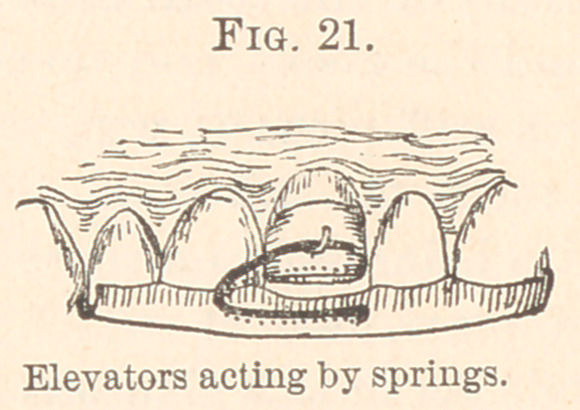


**Fig. 22. f4:**
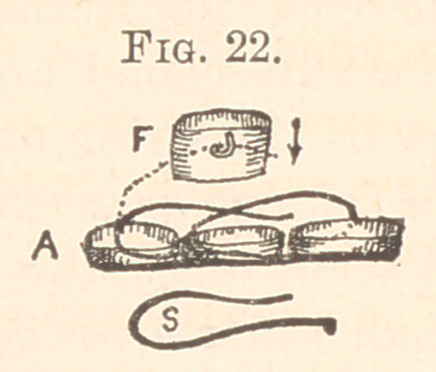


**Fig. 23. f5:**
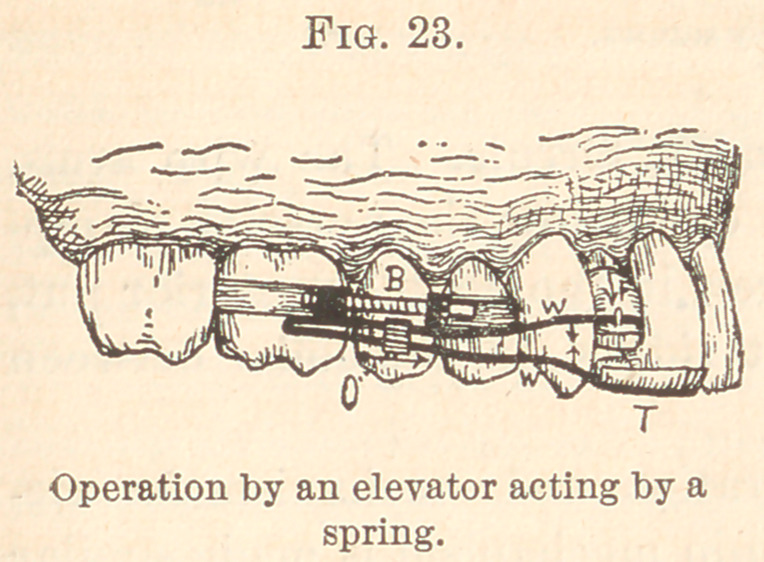


**Fig. 24. f6:**
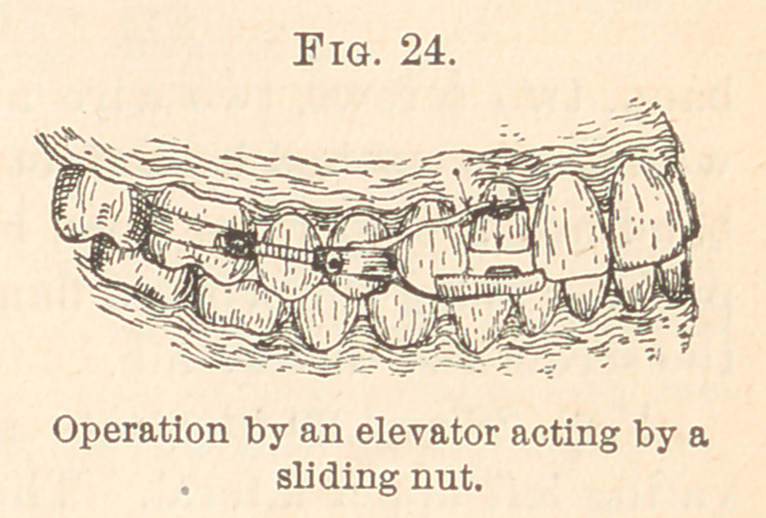


**Fig. 25. f7:**
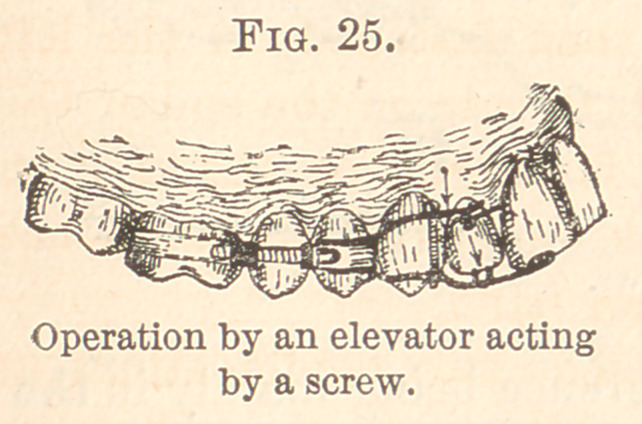


**Fig. 25′. f8:**
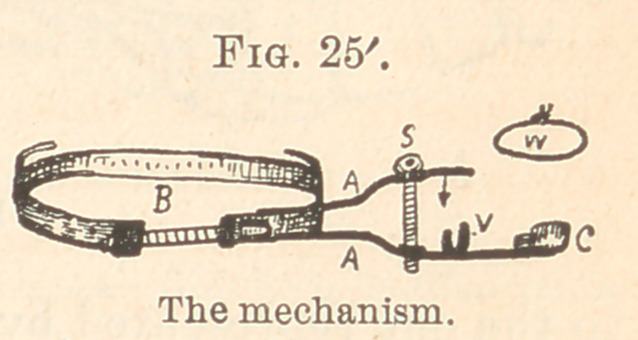


**Fig. 26. Fig. 27. f9:**
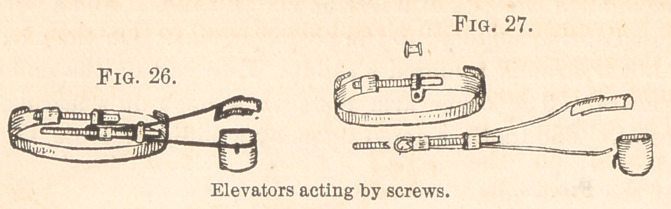


**Fig. 28. Fig. 29. f10:**
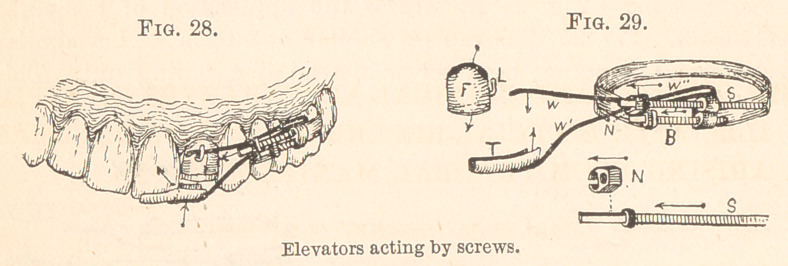


**Fig. 30. f11:**
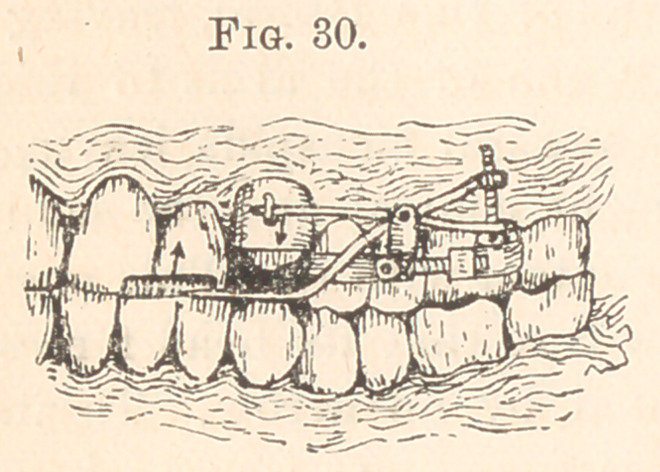


**Fig. 31. f12:**
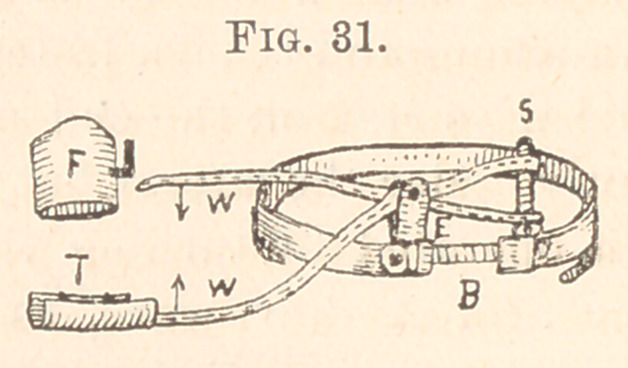


**Fig. 32. Fig. 33. f13:**